# School closure in response to epidemic outbreaks: Systems-based logic model of downstream impacts

**DOI:** 10.12688/f1000research.23631.1

**Published:** 2020-05-12

**Authors:** Dylan Kneale, Alison O'Mara-Eves, Rebecca Rees, James Thomas

**Affiliations:** 1 Department of Social Science, UCL Institute of Education, University College London, London, UK

**Keywords:** School closure, pandemic, logic model, conceptual framework, COVID-19, evidence synthesis, novel coronavirus

## Abstract

**Background:** School closures have been a recommended non-pharmaceutical intervention in pandemic response owing to the potential to reduce transmission of infection between children, school staff and those that they contact. However, given the many roles that schools play in society, closure for any extended period is likely to have additional impacts. Literature reviews of research exploring school closure to date have focused upon epidemiological effects; there is an unmet need for research that considers the multiplicity of potential impacts of school closures.

**Methods:** We used systematic searching, coding and synthesis techniques to develop a systems-based logic model. We included literature related to school closure planned in response to epidemics large and small, spanning the 1918-19 ‘flu pandemic through to the emerging literature on the 2019 novel coronavirus. We used over 170 research studies and a number of policy documents to inform our model.

**Results: **The model organises the concepts used by authors into seven higher level domains: children’s health and wellbeing, children’s education, impacts on teachers and other school staff, the school organisation, considerations for parents and families, public health considerations, and broader economic impacts. The model also collates ideas about potential moderating factors and ethical considerations. While dependent upon the nature of epidemics experienced to date, we aim for the model to provide a starting point for theorising about school closures in general, and as part of a wider system that is influenced by contextual and population factors.

**Conclusions:** The model highlights that the impacts of school closures are much broader than those related solely to health, and demonstrates that there is a need for further concerted work in this area. The publication of this logic model should help to frame future research in this area and aid decision-makers when considering future school closure policy and possible mitigation strategies.

## Introduction

Closing schools in order to limit the transmission of infection during epidemics has long been a recommended course of non-pharmaceutical action under certain circumstances ^1^. The logic underpinning this policy is clear: school children mix at close proximity for extended periods of time, can have poor hygiene, and can then infect parents, carers and other contacts outside of the school setting. School staff are also at higher risk of infection while in the school environment. Previous evidence has shown that closing schools can have the intended impact of reducing infection rates, although factors such as the timing and length of the closures are likely to be important.

If reductions in rates of infection were the only outcome resulting from closing schools, then their closure would appear to be a powerful weapon to be deployed in epi/pandemic situations. However, the decision to close schools is not straightforward. Firstly, it is not necessarily a binary decision; there are situations where partial closure of schools may be possible/essential or, likewise, the closure of, for example, secondary but not primary schools. Secondly, closing schools can have many downstream impacts which may result in unintended and undesirable outcomes, making the decision as to whether (or when, or how) to close schools, and how long for, much more complicated than it might first appear.

Particularly undesirable impacts can include a reduction in healthcare staff availability, just when their presence may be most important, if parents then need to stay at home to look after children. Other members of a family, such as grandparents, may be called upon to take care of children while parents are at work and, given that higher age groups may be more at risk (as is the case with COVID-19 ^2^, the disease caused by the 2019 novel coronavirus) school closures may result in increased infection rates in the ‘wrong’ population groups. While some might welcome the opportunity not to attend school, negative consequences for children might include poorer nutrition, due to missed school meals, and increased exposure to dangerous home environments. School closures are likely to have an impact on children’s educational progress, disrupting the delivery of teaching and assessment, and having potentially far-reaching consequences in the case of long-term closures. Of course, there may be some unintended benefits too, such as improved parent-child relations, reduction in anxiety for some students, or the opportunity to focus learning where needed, but these will be idiosyncratic and very much dependent on the home situation.

Determining the appropriate policy when considering school closures thus requires consideration of a complex range of possible outcomes, which may differ between epi/pandemic scenarios. The 2019 novel coronavirus pandemic has highlighted that, despite many nations having pandemic preparedness plans, the downstream consequences and side effects of school closures are much greater than anticipated. As a result, there has been a collective scramble to address those impacts that are being identified in a reactive – rather than proactive – way. To be better prepared in the future, we need to have a more comprehensive picture of the different factors and impacts of school closures.

Logic models (sometimes called ‘conceptual frameworks’) are a useful tool for decision-makers when considering broader impacts from this type of policy decision, and they can aid planning regarding other measures that might need to be put in place in order to mitigate unwanted impacts ^3^. Logic models are tools used to depict assumptions graphically about the chains of processes, activities or events expected to occur during the implementation of an intervention (in this case, school closures), and the way in which these lead to changes in outcomes. Logic models provide an initial set of assumptions of how different intervention components or intervention models are expected to change outcomes, and can be used to develop further sub-research questions to investigate the validity of these assumptions. In addition to being tools for theorising impacts, logic models are also tools for communicating findings, and in this case can provide a visual account of the possible impacts of school closures across different domains.

Our aim here is to develop a systems-based logic model as an aid in theorising the complexity of school closures and the broader context and impacts, and how these interact ^4^. System-based logic models may be particularly useful in accounting for the myriad ways in which different interconnecting components of health, educational, economic, and social systems are impacted by school closures. The model will provide a starting point for theorising whether there are aspects of the context (setting and existing health/social/education infrastructure) or population(s) that are likely to facilitate or hinder the implementation and effectiveness of school closures, and whether there are particular groups likely to be disproportionately adversely impacted. It is also hoped that the concepts identified in the literature will be supplemented by input from a range of stakeholders, leading to the creation of a more complete model of potential impacts of school closures.

## Methods

The development of this logic model followed a systematic plan in order to ensure that: a) it represents the state of current knowledge and thinking on the subject; and b) that it is rooted in empirical research and not solely the prior opinions of the authors. Although we have adopted a systematic plan and attempted to document all decisions to enhance the transparency of our process, this is not a formal systematic review.

### Searching for evidence

The aim of finding research to inform the development of a logic model is to enable the team to develop a comprehensive picture of the concepts – and the relationships between them. We do not need to find every paper on the subject, but simply a sufficient range of documents to enable us to create a complete model. Thus, the aims of searching differ here from a typical systematic review, in which we would aim to find as many relevant papers as possible to increase precision and reduce (statistical) uncertainty around a given outcome. Given the aim of searching for logic model development, we used ‘snowballing’ methods to locate relevant papers, beginning with a set of known records and moving out from them in a systematic way. This enabled us to refine the logic model iteratively as new papers were discovered and to maximise the efficiency of the search.

As the context of this research is the 2019 novel coronavirus outbreak, we began with a set of nine records for published papers included in a review of school closure and management practices during coronavirus outbreaks ^1^. We used the Microsoft Academic dataset (updated date: 30 January 2020) to locate and retrieve, in addition to the set of nine: all the records in the bibliographies of these papers, all records that cite those papers, and all records that Microsoft Academic ‘recommends’ as being ‘related’ to those records. We then filtered this list to identify the records that mentioned “school” and “closure” in their titles or abstracts.

We conducted supplementary searches to broaden the representation of disciplines and service sectors in our mapping. These included: title- and abstract-focused searches of the educational, psychological and social services literature via the ERIC, PsycINFO and ASSIA databases (see *Extended data* ^5^); an online hand search (27/03/20) of records containing the word “school” within the Cambridge University Press journal ‘Disaster Medicine and Public Health Preparedness’; searches using Google Scholar for studies that themselves cite selected included studies (studies with implementation, policy and/or attitude as a term in the paper’s title). Several policy documents were also included ^6, 7^.

### Screening and coding studies

Screening and coding the studies occurred in parallel. Studies were excluded based on their titles and abstracts if they were:

- not focussed on school closures,
- not focussed on planned school closures, or
- focussed on unplanned school closures not related to pandemics.

Records were flagged as key papers if they were identified (from title and abstract) as having good breadth and depth of the study and a potential for helping us to identify new concepts.

At the same time, we conducted an initial examination of how each paper contributed relevant concepts to the model by coding terms contained in the title and abstract. The codes were developed as we progressed through the list of records; they were not developed *a priori* as we wanted the evidence to drive the model.

Coding reflected (i) the type of intervention (i.e., form of school closure and whether there were concurrent interventions); (ii) impacts (short and long-term, societal and individual outcomes); (iii) context, including the reason for closure (e.g., type of pandemic) and location (country/countries); and (iv) potential moderators. The broad study design was also coded at this stage.

Importantly, we decided to not code extensive information about the preconditions of the school closures. This is because we want to draw more attention to the huge range of possible outcomes and impacts that could result from school closures. Where the preconditions (e.g., features of the virus itself) were presented as potentially moderating or mediating the effect of the intervention on the impacts, we recorded that information as a factor. This decision has inevitably constrained the scope of our model to make the closure of the schools the starting point of the model, although clearly much has to happen beforehand to determine the introduction – and form – of the school closure policy.

We then retrieved the full-text documents of the key papers to check for information not already captured at the title and abstract stage. A number of studies were deprioritised as reporting studies where the concepts had already been identified (i.e., we had reached conceptual saturation), mainly reflecting simulation studies that modelled the hypothesised impact of school closures on viral transmission rates, or peripheral studies where the impact of school closures was not a focus of the study. Full texts were not obtained for these deprioritised studies.

### Developing the model from the codes

Working with the extracted codes, and Miro software, we sorted the impacts identified in the literature into logical chains, drawing as much as possible from the information contained within the studies themselves to theorise the sequence of outcomes. This stage was largely conducted in collaborative virtual meetings between the authors, in which we discussed the positioning of the codes that we had identified and added any unrepresented factors (see below).

The codes were arranged according to broad domains (e.g., impact on children’s education), and chains of outcomes were hypothesised in some cases to help identify more distal outcomes ^3^. Additional factors that could moderate the impacts of school closures including broader context and setting factors only appear as a list in the model due to the complexity of relationships. We also considered whether more complex relationships between outcomes needed to be depicted (particularly vicious or virtuous cycles but also instances of conjunctural causation). Working collaboratively, the team met at several points to discuss the evolving logic model.

A number of factors were not explicitly represented in the literature, the most prominent gap being impacts on children’s educational achievement and development. We supplemented the model with additional hypothesised factors, shaded in a different colour (green), to indicate that these were not explicitly mentioned in the included literature. These are largely drawn from the media, policy documents, or anecdotal evidence (e.g., the experience of the researchers). These were added in acknowledgement that not all academic papers have considered all possible impacts (indeed, this concern was one of the motivating factors for undertaking this work).

Although the school closure intervention is the starting point for our model, we decided that it was easier to view the model with the intervention in the centre and the proposed impacts emanating out from there. It is important to bear this in mind when viewing the model: the ordering in the diagram does not reflect temporal or causal ordering, nor does it indicate order of importance—we did not collect data on these aspects. The positioning of the impacts emanating from the centre is largely determined by aesthetics and readability, such as by placing linked concepts near each other to minimise arrows stretching across the model.

In order to develop our model around potential impacts of school closures, and in line with recommended practice in constructing logic models ^8^, we are publishing an early version of the model for input across a range of stakeholders and peer reviewers. On the basis of this input, a second version of the logic model will be published, following an iterative model, and we hope to investigate the evidence underlying the outcome chains further. We expect that stakeholder involvement will help us to consider further impacts, drawing on the principles of ‘dark’ logic models through incorporating the expertise and insight of individuals or groups from different contexts within the UK (and beyond) ^9^.

## Results

### Identifying relevant research

**Figure 1.**
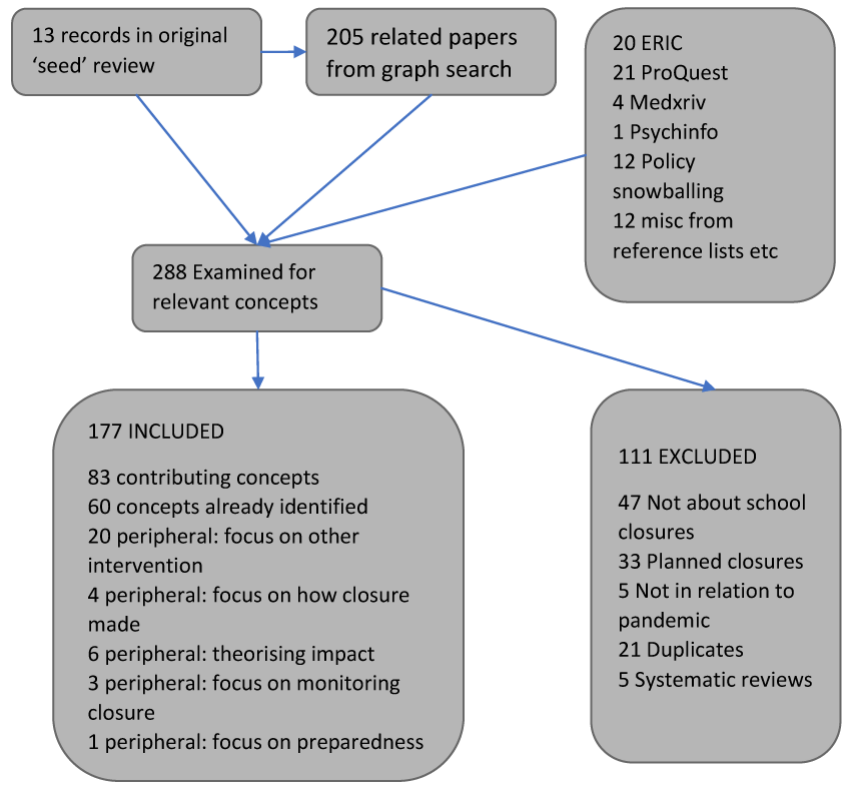
Sources of papers used to identify concepts.

Our initial search of Microsoft Academic resulted in a set of 205 unique records to examine. We then added additional records from supplementary searches of databases, reference lists (including systematic reviews), and policy documents. When the additional records are included, we had 288 unique papers to utilise to identify the concepts present in our map ( Figure 1). A total of 177 papers were used in some way (though some contained concepts we had previously identified and were not examined in detail) and 111 were not relevant. (See *Underlying data* ^5^ for a list of the 177 references.)

### The logic model

**Figure 2.**
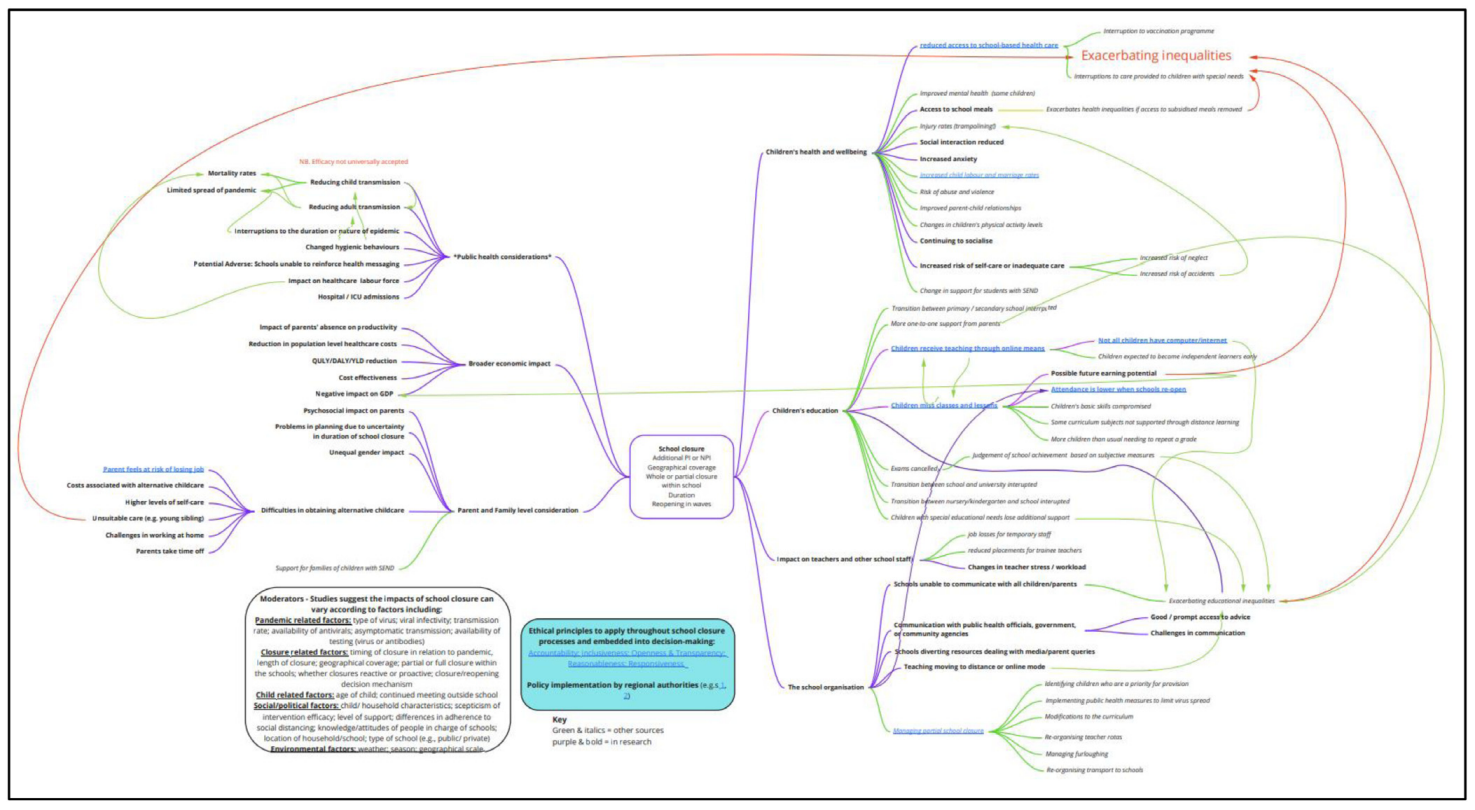
Overall conceptual framework.

The resulting logic model (see Figure 2) contains more than 100 concepts that are organised into seven higher level domains: children’s health and wellbeing, children’s education, impacts on teachers and other school staff, the school organisation, considerations for parents and families, public health considerations, and broader economic impacts. As mentioned above, the positioning of the higher-level domains in the model is not designed to indicate their importance or temporal ordering. In addition, a large number of effect moderators were identified, organised under five headings: pandemic related factors, closure related factors, child related factors, social/political factors, and environmental factors. The final component in the logic model is the presence of the ethical principles that apply throughout school closure processes and which are embedded in decision-making.

The graphical depiction of the logic model is shown in Figure 2. As this is a large image, we have shared a link to the Miro board where an interactive version can be found, here: https://miro.com/app/board/o9J_kuvvCJo=/. Concepts and relationships that were identified in the research literature are coloured in bold with purple connecting lines. Concepts identified through team discussion and their engagement with wider literature (e.g., stories in the media) are depicted in italics with green connectors.

***Study characteristics.*** The logic model was developed from concepts identified in 177 included studies. These considered the impacts of school closures, either in isolation or along with other pandemic mitigation strategies. The majority of papers considered the impacts of school closures related to the pandemic H1N1/09 virus in 2009 (also known as swine flu) (n = 74 papers) ^10, 11^. This strain disproportionately affected children and younger adults ^10^, and school closures were part of pandemic mitigation strategies across a number of different countries. However, the decision to close schools during this pandemic was discretionary in several settings and dependent on the number of cases in the school or the immediate locale (for example ^12^). as well as other factors, and often not part of nationwide pandemic control measures. Furthermore, the closure of schools tended to be for a much shorter period than in the current 2019 novel coronavirus pandemic, for example for a week ^13^, although there may have been recurring closures in some settings (for example ^14^).

Other papers considered the impact of pandemic flu more generally (n = 60 papers) including the 1919 flu pandemic (n = 3 papers), or considered other forms of infectious disease or pandemics (n = 24, e.g., Hand, Foot and Mouth disease), or the response of schools to localised outbreaks of cases within schools (n = 4). A smaller number of papers considered recent school closures in the context of recent outbreaks including severe acute respiratory syndrome in 2002-2004 and Middle East respiratory syndrome, another form of coronavirus outbreak identified in 2012. As with H1N1, closure decisions were localised and reactive or discretionary in some settings (for example ^15^), although they were more widespread and part of public health policy in others (for example ^16, 17^); however, closures tended to be shorter than those being implemented in the 2019 novel coronavirus pandemic (for example ^17^). Finally, six studies were identified as being focussed on the2019 novel coronavirus pandemic from different settings including China (n = 3), Hong Kong (n = 1) and the United States (n = 2). As these are studies considering closures currently in progress, they may not fully capture the potential short and long-term impacts of sustained periods of school closures.

Overall, the composition of the included studies means that the concepts in the logic model are generally drawn from studies on short-term school closures that may not be implemented as nationwide policy. Such scenarios are vastly different to the 2019 novel coronavirus pandemic, which has seen the introduction of widespread and long-term non-pharmaceutical interventions, of which school closures is just one. This makes obtaining input from a wide range of stakeholders to supplement the concepts represented in the logic model all the more important.

We will now briefly outline the main concepts in the logic model, beginning with the intervention, then the seven higher level domains, then the moderators, and finally the ethical frameworks.

**Figure 3.**
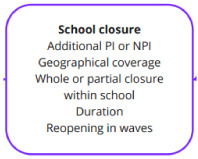
Logic model section: school closure.

***The intervention: school closure.*** The form of the school closure—that is, the way that the policy intervention was implemented—was seen to vary substantially between epidemics and by country/region/jurisdiction ( Figure 3). In most cases, the closure of schools was one of a number of pharmaceutical interventions (PI, such as vaccine) and or non-pharmaceutical interventions (NPI, such as public transport restrictions and social distancing of confirmed cases). This is very important to bear in mind, as the presence of pharmaceutical interventions such as vaccines and treatments may greatly reduce the need for NPIs, while the converse is also likely. Further, in a complex system of NPIs over a long period of time, the relative costs and benefits of all measures need to be considered. For example, some of the impacts covered in the domains below are likely to be exacerbated in a stringent social distancing scenario (“lockdown”), but might be less problematic if other social distancing measures are relaxed.

The geographical coverage of the closure was also quite variable; in some studies, only schools with confirmed cases closed (referred to as reactive closure), whereas others involved a school district or wider region (e.g., a state or country) and could be considered proactive closures (closing even if the school has not had a confirmed case within its student population).

There are various models of school closure. Whole school closures are the most intensive, but other partial options indicated include just some types of school (e.g., just nurseries), targeted class or grade closures within a school, or alternating class attendance on different days of the week or alternating weeks.

The duration of the closure is likely to be one of the biggest factors in determining the impacts, and some impacts may not be relevant for very short closures. Very short-term closures, for example, are unlikely to have much impact on educational achievement or future earning potential, although this is hypothesised rather than detail we extracted from the papers. Linked to duration, some papers mentioned reopening the school in waves—i.e., subsequent reclosing of a school after a period of reopening, usually in response to new confirmed cases or additional peaks in the pandemic.

In summary, the policy intervention, school closures, took many forms. Although it was not within the scope of this work, we suspect that the form of the school closure is likely to change the logic model quite substantially, with some impacts only being observed in longer term closures or when broader social distancing measures are in place.

***Higher-level domains***

**Impacts on children’s health and wellbeing**

**Figure 4.**
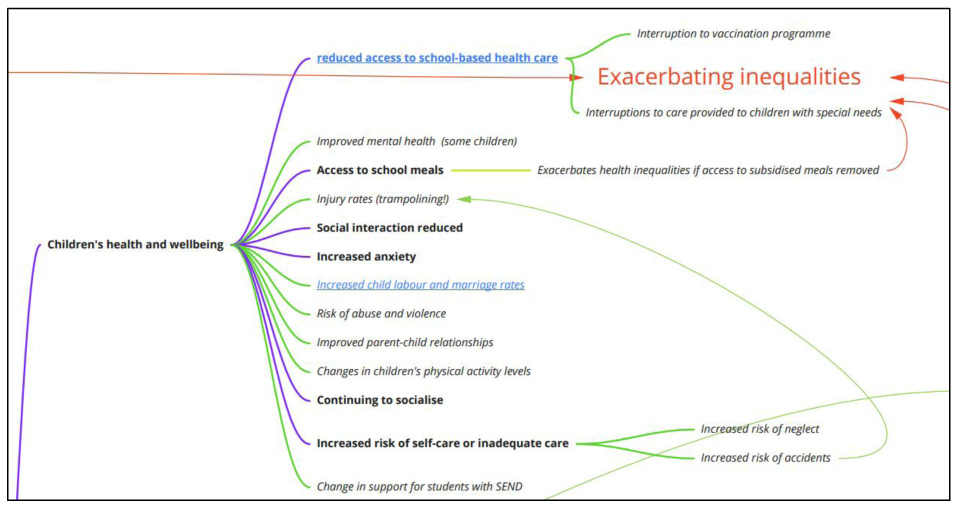
Logic model section: impacts on children’s health and wellbeing.

Although parents may be concerned about the impact of school closures on their children’s health and wellbeing, there were relatively few concepts identified from studies that provided much indication of the nature of these impacts ( Figure 4). Studies had considered that closure of schools also entailed losing access to school meals, which we hypothesised would exacerbate health inequalities if access to subsidised meals was removed, as well as broader forms of inequality. The literature had considered that closing schools would disrupt and reduce children’s social interactions, although some studies also suggested that some children would continue to socialise. Such impacts are likely to be affected by the presence or absence of additional social distancing measures, and how stringently they are enforced or adhered to.

Closing schools could impact on children’s health, through increasing levels of anxiety, as well as impacting on access to school-based health care, which in turn was hypothesised to interrupt public health strategies such as vaccination programmes for other diseases (e.g., MMR) and to interrupt care provided to children with special health needs (e.g., asthma). Some studies considered increasing levels of self-care or inadequate care arising from the closure of schools, which in turn was hypothesised by the team to increase the risk of neglect or accidents occurring, the latter in turn being linked with a risk of injury. Given that most of the included studies examined short-term closures, the risk of these factors occurring may be different in the context of the 2019 novel coronavirus pandemic where school closures may last several months, and these potential impacts should be considered in light of the list of moderators (discussed below).

The logic model also contains a number of impacts that were not considered within the research literature, but instead were proposed by our team, and included potential serious negative impacts (e.g., increased risk of abuse and violence) as well as some positive impacts (improved parent-child relationships and improved mental health for some children). These additional impacts are represented in italics with green arrows in the diagram. Finally, although not based on academic literature, school closures may increase the risk of child marriage and child labour, a concern flagged by the United Nations ^7^. Clearly school closure is going to be experienced differentially across existing lines of inequality, and although some of the hypothesised pathways are represented here, the publishing of this model for public input is expected to enhance our understanding of the impacts of school closure on children’s health and wellbeing.

**Impacts on children’s education**

**Figure 5.**
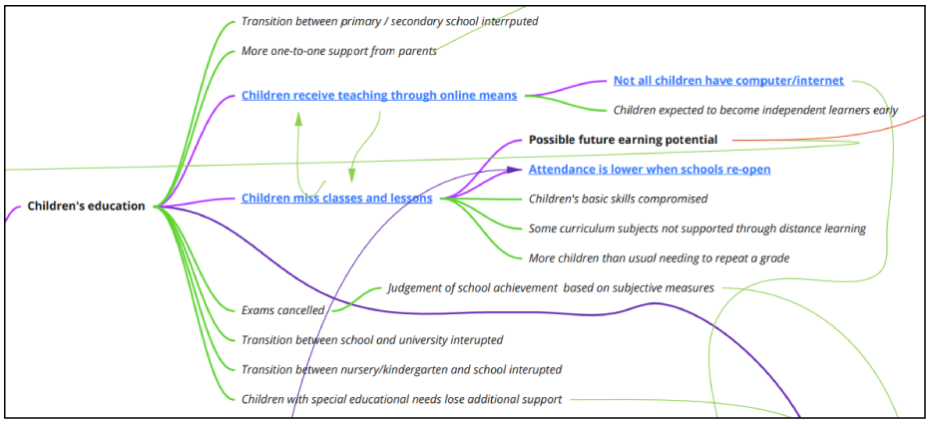
Logic model section: impacts on children’s education.

Relatively few concepts were identified in the literature that indicated the impact of school closures on children’s education, a likely artefact of the short duration of closures examined in extant studies ( Figure 5). Studies considered children missing classes and lessons, which may impact on their future earning potential, as well as disrupting attendance patterns once schools reopen. If the children’s mode of education shifts to online once schools close, this might exacerbate inequalities where children are unable to access suitable equipment at home and may mean that some children are particularly vulnerable to missing classes and lessons. A hypothesised implication of shifting education to online modes is that children are expected to become independent learners earlier than expected, and earlier than is developmentally suitable.

Hypothesised consequences of children missing lessons are that the development of their basic skills (e.g., literacy, numeracy) may be compromised, and children may not be taught subjects that are more challenging to deliver through distance learning (e.g., Music or Design & Technology). Although there has been widespread concern in the media about the cancellation of exams ^18^ due to the 2019 novel coronavirus, this has generally not been explored in literature on pandemic-related school closures. Similarly, we can only hypothesise that school closures will impact on transitions between different settings (nursery/kindergarten to primary school; primary to secondary; secondary to college or university). We also hypothesise that children with special educational needs may lose additional support ordinarily received within educational settings, exacerbating educational inequalities. In contrast to other domains, the impacts of school closure on children’s education and potential ways of mitigating these impacts are mainly hypothesised, and the concepts represented tended not to be drawn from the literature.

**Impacts on teachers and other school staff**

**Figure 6.**
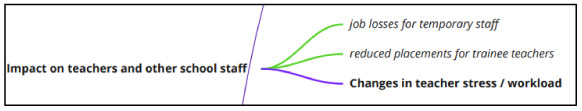
Logic model section: impacts on teachers and other school staff.

We found this area of impacts also to be a relatively unexplored one in the research literature. We found research papers that discussed impacts on teachers in terms of increased workloads during school closure and stress in the run up to closure. Concepts not found in the included research or policy literature but evident elsewhere (see italics, Figure 6) include concerns over job losses for school staff with less secure contracts and interruptions to teacher training.

**Impacts on the school as an organisation**

**Figure 7.**
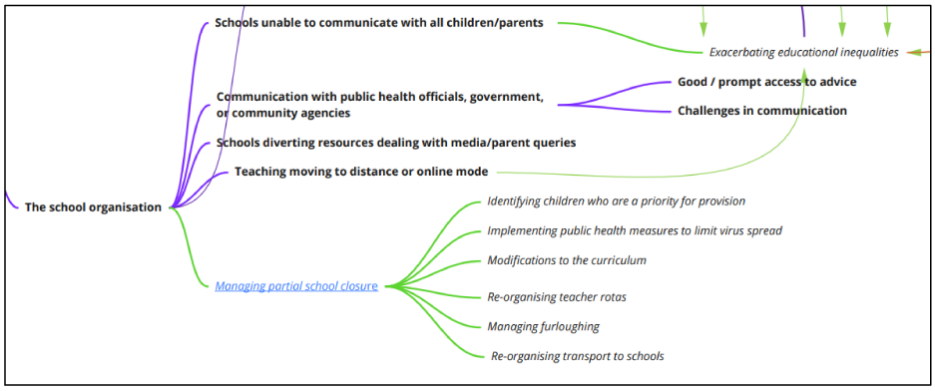
Logic model section: impacts on the school as an organisation.

We found reference in research papers to changes in teaching provision, in terms of moves to teaching at a distance ( Figure 7). Studies also referred to interactions with bodies external to the school such as those with responsibility for public health or social care, as well as communications with parents. The latter of these explored the challenges of handling large numbers of queries, and challenges in reaching all parents with communications. There was reference to resources being diverted to deal with additional communication work.

Aspects considered where schools are only partially closed were found only in the policy literature (and so are identified in italics in the logic model). These included the identification of children who should be encouraged still to attend (e.g., those of key workers, or children judged vulnerable by social care authorities); the implementation of procedures to promote social distancing and hand-washing; the reorganisation of the curriculum in order to prioritise safeguarding; the reorganisation of teaching staff rotas (e.g., to cover holiday periods, absent staff, and alternative teaching settings); the managing of furloughing (e.g., for temporary staff); and aspects of student transport.

**Parent and family level considerations**

**Figure 8.**
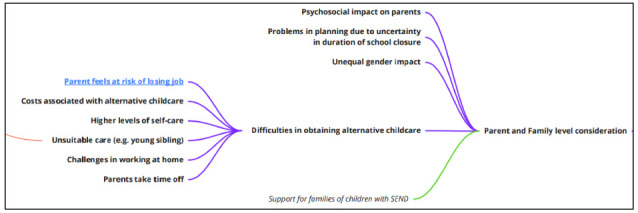
Logic model section: parent and family level considerations.

The research literature explored the potential for impacts on parents and families in multiple areas, with those associated with obtaining alternative childcare at the fore ( Figure 8). This conceptual area, as with impacts of school organisation and school staff, tended to be determined by researchers in their setting of questionnaire items but were also sometimes contributed directly by study participants who had been asked open-ended questions.

Here, parents may find themselves needing to juggle a need to work from home with looking after their children – and possibly taking a role in their education at the same time. In situations where the parent cannot work from home, they may need to take time off which, in extended periods of school closure, may be unpaid, and they may be at risk of losing their job if they cannot work. Sometimes, parents will arrange alternative childcare which might be expensive, increasing household expenditure at a time of economic uncertainty.

There is also a risk that alternative childcare provision might be unsuitable (for example, there can be increased risk of injury where an older, but nevertheless young, sibling is called upon to look after their younger sibling(s)). Pre-existing employment insecurity may be magnified in these situations, and households with limited disposable income will be less able to pay for childcare; thus, disadvantaged groups are more at risk than others, and gaps and gradients in inequalities might be increased in some situations. Of relevance here are potentially unequal gender impacts, with women more likely to take on increased childcare responsibilities, and single-parent households likely to find the situation more challenging. Uncertainty in the duration of the school closure can make planning difficult, which might also have economic and mental health consequences for those attempting to make alternative working and childcare arrangement without knowing whether they are planning for several weeks, or months. Dealing with the above can have a detrimental psychosocial impact on parents and carers.

**Broader economic impacts**

**Figure 9.**
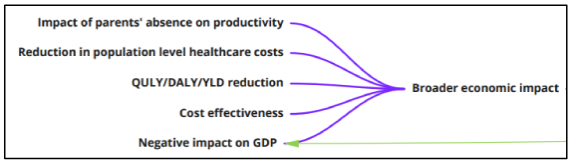
logic model section: broader economic impacts.

Researchers have examined possible broader economic impacts resulting from school closures ( Figure 9). Given that the main aim of closing schools is to reduce infection rates in epidemic/pandemic situations, the *cost effectiveness* of this is considered in a range of ways. Some papers carry out traditional health economic analyses, examining impacts in terms of quality-adjusted life years, disability-adjusted life years and years lost due to disability. Others examine the impact of parental and carer absence on economic productivity and how this might impact negatively on GDP. Other analyses consider how the downstream impact of school closures might result in a reduction in population level healthcare costs.

**Public health considerations**

**Figure 10.**
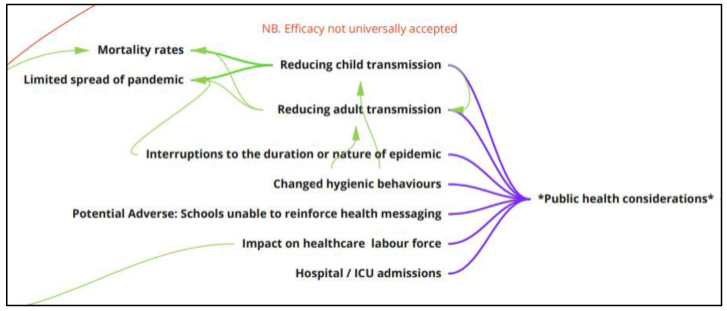
Logic model section: public health considerations.

Most of the papers that we identified focused on the public health considerations of school closures ( Figure 10). The observed or predicted reduction in the transmission rate was by far the most common issue considered. Some of the papers assessed the likely reduction in transmission rate under certain conditions, such as the infectivity rate, combination with other interventions (e.g., availability of a vaccine or other social distancing measures), or duration of the closure. Some simulation (modelling) studies questioned the cost-effectiveness of school closures in certain scenarios. The consideration of moderators is likely to be very important in understanding the impact of school closures on virus transmission rates.

Other public health considerations were mentioned in the literature. For example, mortality rates were often considered in terms of the relevant sector of the population that might see an improved mortality rate. Some studies noted that school closures may affect mortality rates in age groups beyond the school age via the reduction in transmission rates outside of the school setting.

Changes to the spread of the virus were also considered. In particular, limiting the spread of the pandemic to other geographical areas beyond the initial observed site/s and interruptions to the duration or the nature of the epidemic (including the timing of peaks of viral infection) were mentioned. Unsurprisingly, the impact on the health care service was considered in some studies. This included discussion of hospital and intensive care unit admissions, the purchasing of virus-relevant medications, and visitations to GPs.

The removal of children from a school setting was proposed to have a potential adverse impact because schools are then unable to reinforce health messaging. There may also be changes in the hygienic behaviours of children, such as hand washing, as they would have in the school, although this could be compensated by changes to hygienic behaviours in the home.

One public health impact is linked in our model to other impacts discussed above under parent and family considerations and broader economic impacts. This is seen as stemming from a reduction in the healthcare labour force when frontline healthcare workers (doctors, nurses, etc.) need to care for children at home and are unable to work or can only work reduced hours. There is a hypothesised consequent increase in the overall mortality rate (not just due to the pandemic), as health service demand exceeds capacity.

***Mediators and moderators.*** Every study that we looked at either directly or in passing considered potential moderators or mediators of the impacts of school closures. These are factors that might change the impact that school closures have on a given outcome. The relation between the school closures intervention and key outcomes is likely to be very influenced by a range of factors working together in a complex relationship. Because of the complexity of these relations, we have not generally tried to include the factors in the pathways of the logic model; instead we are listing them as factors to consider when viewing the relation between school closure and proposed outcomes. We have grouped the factors into similar types of concepts.

- **Pandemic-related factors**, which are those that pertain to the virus itself. These include the type of virus (e.g., specific influenza strains); viral infectivity (or virulence, the ability of the virus to establish an infection); transmission rate (the speed at which new transmissions occur, although this sometimes appears to be used interchangeably in some papers with reproductive ratio); availability of antivirals or vaccines; whether the virus exhibits asymptomatic transmission; availability of testing (virus or antibodies). There are likely to be others not observed in our sample of studies, including the infectious period (how long an infected person is able to transmit the infection) and mode of transmission (airborne, faecal-oral, vector-borne, etc).
- **Closure-related factors,** which are those that relate to the way in which the closure is implemented. This overlaps with the features of the intervention itself, which are listed in the central box of the model. Closure-related factors may include the timing of the closure in relation to pandemic (e.g., before or during the peak of infections); length of closure; geographical coverage (one or small number of schools, a whole school district, city, region, nation); partial or full closure within the schools (e.g., only certain grades, or certain students); whether closures are reactive (closure after a confirmed case) or proactive (closure before confirmed case); closure/reopening decision mechanism (e.g., is it discretionary or mandatory, and who decides).
- **Child-related factors**, which are those factors relating to the students whose schools have closed. These include the age of the affected children and whether they continue to meet or socialise outside of the school setting.
**Social/political factors**, which are those that relate to the social or political context in which the school closures take place. These include child or household characteristics (e.g., socioeconomic status); scepticism of intervention efficacy (i.e., doubt that the school closure will have the desired effect or the benefits outweigh the costs); level of support; differences in adherence to social distancing; the knowledge/attitudes of people in charge of schools; the location of household/school (e.g., rural versus metropolitan, areas of social deprivation); and type of school (e.g., public/ private).
**Environmental factors**, which are those that relate to the environmental context of the pandemic. These include the weather, season, and geographical scale of the infection’s reach

***Ethical and local policy implementation factors.*** School closures raise a number of questions for decision-makers around the way in which they should be implemented and how they can be implemented ethically and equitably. Closure decisions made within small areas (e.g., local or regional districts) raise particular considerations with regards to implementation, and may require considering developing policies across different policy domains. One study drew on an existing framework for decision-making during pandemic preparation ^19^ that includes processes to be implemented, and values to be considered, in ensuring an ethical response to pandemic preparedness. The study ^20^ adapted the original framework to examine the implementation of decisions around school closures as a result of H1N1 pandemic, and considered processes including (i) accountability, openness and transparency (decisions are communicated and open to scrutiny); (ii) inclusivity (decisions are made with the views of stakeholders in mind); (iii) reasonableness (decisions are based on evidence, principles and values); and (iv) responsiveness (decisions are revisited on the basis of new information). In addition, these were supported by values including (i) a duty to provide care; (ii) protection of the public from harm; (iii) stewardship; (iv) solidarity; (v) privacy; (vi) individual liberty; (vii) equity; (viii) proportionality; (ix) reciprocity; and (x) trust.

## Discussion

### Summary

The logic model presented here presents a summary of the concepts considered by studies on pandemic-related school closures, grouped into domains of impacts. Many of the public health impacts considered in the literature involve examining reduction in viral transmission rates, lowering mortality rates, and limiting the spread of the pandemic, suggesting that the literature has tended to explore the positive epidemiological impacts of closures. Other domains, such as the impact on parents and families, children’s health and wellbeing, and children’s education, are largely composed of concepts relating to the negative impacts of school closures. Similarly, many of the broader economic impacts considered by the literature involve negative impacts on productivity and GDP, as well as consideration of cost-effectiveness of closures. Concepts around the impact on the school as an organisation and on teachers reflected some of the impacts of (sudden) pandemic-related closures and the resulting disruption on school functioning and teacher workload. Some of the impacts considered involved longer-term impacts of closures, such as the impact of the loss of education on children’s future earning potential. Across several of the domains, many of the concepts identified from the literature are hypothesised to exacerbate inequalities, including health and educational inequalities, if left unchecked.

Due to the nature of the extant evidence base, which mainly reflected evidence on shorter-term, often reactive or discretionary, school closures, few of the concepts reflected interventions or steps to mitigate the impact of school closures. Some of the concepts did reflect potential adaptations to the closure of schools, notably the movement of learning to online means, a concept that emerged in studies on the 2009 H1N1 pandemic. However, in turn, there was also consideration of how movement of teaching online could exclude those children without computers or reliable internet access, thereby exacerbating inequalities in the impacts of the pandemic among children from poorer socioeconomic backgrounds.

Some of the studies examined the implementation of school closures. Although these were of peripheral interest given our primary concern around mapping and hypothesising the impacts of school closure, these did nevertheless illuminate the challenges of making school closures decisions. In addition, some that were more focussed on implementation also provided frameworks around how the impacts of school closures could be understood from an ethical perspective ^20^, providing potential insights into the types of process and value considerations that might be useful to incorporate in the design of policies and interventions to mitigate the potential negative impact of school closures.

### Strengths and limitations

This logic model represents a map of concepts from studies identified through a rapid systematic search. The concepts were mainly drawn from those mentioned in the title and abstract of studies, and only those studies that appeared to have greater breadth and depth were selected for full text review to help to identify new concepts. While this is a pragmatic strategy, suitable to our purpose of identifying concepts but not synthesising findings, it does mean that some more marginal concepts may have been missed from the logic model. Similarly, although the model is based on a systematic search with supplementary searches of policy documents, many of the steps that could have been conducted in duplicate in a systematic review (e.g., screening and coding of studies) were instead conducted by a single researcher because of the rapid nature of the study. In addition, although a concept may have been considered within a study, this does not give an indication as to the weight of evidence supporting that concept.

Although the model considers some elements in the implementation of school closures, the model does not explicitly represent the rationale or triggers for closure (or non-closure). This includes those factors that influence the decision to close (e.g., legal or jurisdictional factors), levels of public support, the type of closure policy set in place (e.g., all or some schools, or parts of schools, closing), or the adherence to closure. These factors were beyond the direct scope of the model but are likely to further moderate the impact of closures. They may also be important elements for decision-makers to consider in developing policy around the re-opening of schools.

The map is reflective of the global literature, although there are likely to be heightened challenges in closing schools in a number of different contexts and scenarios. While these factors, which may moderate the severity of impact of closure are represented in the model, few studies were explicitly focussed on any of these groups or settings. For example, few studies explored the impact of closures in the case of special schools, or focussed on children with particular educational or social needs, or focussed on disadvantaged groups. Similarly, although the logic model is representative of the available global literature on pandemic-related school closures, studies exploring the impact of school closures on children in the Global South were largely absent.

As outlined earlier, most of the studies examined the impacts of short-term school closures. Although most of the concepts outlined are applicable to the current context, the implementation of longer-term school closures with uncertain end dates may introduce a number of new impacts that are not fully represented in the model. Further input is welcome to supplement the concepts represented, and we intend to engage with both reviewers as well as a broad range of stakeholders openly and transparently during the peer review process in creating a revised and expanded model. This iterative approach represents a strength of the study, and obtaining the input of stakeholders in this way is a key recommended process in the development of a logic model to strengthen the salience of the model and its value in subsequent research activities ^21^. Different stakeholders (e.g., evaluators, policy-makers, community leaders, parents, teachers, children) tend to hold different views and understandings, which are useful to incorporate when dealing with the uncertainty and complexity in pandemic emergencies. We hope that this involvement can help us in supplementing the range of concepts identified, provide a useful challenge to the assumptions we have made, ensure that a diversity of perspectives are represented, help us in identifying how contextual factors may amplify or dampen the impacts of school closures, and enhance the usefulness of the model ^21^. The revised logic model is intended to be used as a framework to guide further research, as well as a tool for policy-makers to consider the range of different impacts that follow from school closures.

While the model represents some of the downstream impacts resulting from school closures, we know little about the long-term impacts, and the model is underrepresented in a number of domains. Similarly, there were few studies employing quasi-experimental methodologies to understand the causal impacts of pandemic-related closures on outcomes other than health. In the absence of evidence based on a range of methods and considering a breadth of impacts, both long-term and short-term, it is also questionable whether pandemic preparation plans that are being deployed in the 2019 novel coronavirus pandemic also consider a sufficient range of outcomes, and by extension, have developed strategies on how these could be mitigated.

The model is designed to cover as many impacts as we could identify, but it needs to be emphasised that not all impacts will be observed in all scenarios, or to the same extent. For instance, short term closures or those without concurrent additional social distancing measures are likely to mean some impacts are irrelevant and could be dropped from the model for that scenario. In any scenario, the moderators and mediators might reduce some impacts to negligible levels. And even within the same scenario, different individuals will experience the impacts in different ways. We have not made any attempt to quantify the strength of the relationships partly because of the extreme complexity and the idiosyncratic nature of the pandemics, the closure implementations, the moderating factors, and the individual responses to and experiences of pandemic policy interventions. In other words, we present this model as an aid to thinking about what might be important, rather than suggesting that school closures will necessarily lead to any or all of the proposed impacts; such assessments will need to be done with the full contextual information of a given pandemic scenario. 

## Conclusions and future directions

Many papers aim to model the impact of school closure in isolation, rather than as part of a package of social distancing measures. They often conclude that it is difficult to isolate effects on transmission rates, and where these are estimated, they tend to be small. However, identifying the impacts of school closures in isolation is not particularly useful in terms of informing decision-making, since school closures are usually only *part* of a much broader range of measures. Our examination of the range of potential impacts of school closure shows how widespread and wide-ranging they might be. Modelling transmission within its wider context – and including some of the mediators and moderators we have identified – may result in more actionable recommendations for decision-makers.

Part of understanding the wider impacts of school closures entails ensuring a broad range of perspectives are represented within the research literature. Notable within the body of research encountered in developing the logic model was the absence of studies published in psychological, educational and social care journals, among others. Clearly the impacts of school closures are much broader than those related solely to health, and the logic model and its representation of impacts that are considered in the literature highlights that there is a need for further concerted work in this area. This includes setting a multidisciplinary research agenda that represents the perspectives of educational and social researchers, as well as health and epidemiological researchers, in order to broaden our understanding of the impacts of school closures.

Two further revisions to the model will be considered after further stakeholder input. Firstly, we may seek input from researchers who have published on the impact of school closures, to discuss our model and the validity of the underlying assumptions. Secondly, we may consider whether different models are needed for different scenarios of interest to better clarify the concepts and outcomes. For example, we will consider whether different models are required based on the closure of different types of school (e.g., primary vs secondary school) or different patterns or models of closure and potential reopening of schools.

## Data availability

### Underlying data

Zenodo: School closure in response to epidemic outbreaks: Systems-based logic model of downstream impacts. http://www.doi.org/10.5281/zenodo.3780348 ^5^.

File ‘Underlying data - Studies used to develop logic model’ contains the list of references used to develop the model described herein.

### Extended data

Zenodo: School closure in response to epidemic outbreaks: Systems-based logic model of downstream impacts. http://www.doi.org/10.5281/zenodo.3780348 ^5^.

File ‘Extended data - search strings’ contains the search strings used in the literature search performed in model generation.

Data are available under the terms of the Creative Commons Attribution 4.0 International license (CC-BY 4.0).
