# Supplementary material for: School closure in response to epidemic outbreaks: Systems-based logic model of downstream impacts
Source: F1000Res. 2020 May 12;9:352. [Version 1] doi: 10.12688/f1000research.23631.1 (PMC7445561; doi:10.12688/f1000research.23631.1)
Supplement: Peer review history

# Peer review: Reviewer response for version 1

**DOI:** 10.5256/f1000research.26076.r69701

**Published:** 2020-08-24

**Authors:** Marla Petal

**Affiliations:** 1 Save the Children, South Carlton, Vic, Australia

Very timely and valuable contribution. Well presented. 

 Miro presentation is very handy to cover the complex topic. Professional info-graphic design might really enhance differentiating the content for better understanding.

 A couple of things that could be clarified:

 "We also considered whether more complex relationships between outcomes needed to be depicted (particularly vicious or virtuous cycles but also instances of conjunctural causation)."  insider-language... please unpack. 

 "trampolining"?  "continue to socialise" - is that in reference to having some normal interaction (eg bubble group?) 

 + theoretical: some children doing better at home due to avoidance of bullying and other violence against children... 

 Where does this go next:

 #1. A key statement buried in the main text that can be brought out in the discussion: 

 "Although it was not within the scope of this work, we suspect that the form of the school closure is likely to change the logic model quite substantially, with some impacts only being observed in longer term closures or when broader social distancing measures are in place."

 #2. Given that you recognize that a) previous research was on much shorter-term closures b) previous pandemics didn't have the enormous complication of asymptomatic transmission (and you might also mention cost/invasiveness/slow results of testing... )

 I think that it becomes important to open the door on 

 a) the very critical 'implementation and effectiveness of public health measures'... which will need tracking and unpacking (for different age groups)

 b) the variation in interventions emerging which are designed to mitigate many of the impacts.  (eg. see NYC and others continuing to distribute school lunches even though "closed" etc.)

 I really hope that you can take this to next level (next article, perhaps) to do the same for intervention models, because we don't have the classification/language to discuss the huge range of interventions (radio, tv, teacher phone contact, teacher individual and small group mtgs with caregivers or students, peer learning groups, bubble groups, etc..

Is the work clearly and accurately presented and does it cite the current literature?

Yes

If applicable, is the statistical analysis and its interpretation appropriate?

Yes

Are all the source data underlying the results available to ensure full reproducibility?

Yes

Is the study design appropriate and is the work technically sound?

Yes

Are the conclusions drawn adequately supported by the results?

Yes

Are sufficient details of methods and analysis provided to allow replication by others?

Yes

Reviewer Expertise:

Hazard and disaster impacts on education, children's impacts on household safety, evidence-based practice & practice-based evidence for child-centred risk reduction and school safety, school-safety self-assessment.

I confirm that I have read this submission and believe that I have an appropriate level of expertise to confirm that it is of an acceptable scientific standard.

# Peer review: Reviewer response for version 1

**DOI:** 10.5256/f1000research.26076.r69702

**Published:** 2020-08-18

**Authors:** Lisa Pfadenhauer

**Affiliations:** 1 Institute of Medical Informatics, Biometry and Epidemiology, Pettenkofer School of Public Health, Ludwig Maximilian University Munich, Munich, Germany

Thank you very much for providing me with the opportunity to review this very interesting article. This is a very much needed analytical framework which allows researchers and policy-makers alike to structure their thinking around the downstream effects of school closures as widely debated non-pharmaceutical intervention. Please find below a couple of minor comments and thoughts:

**Introduction:**

 Overall, I think the introduction would benefit from more literature, e.g. on the effect of school closures on the course of a pandemic and potential side effects thereof. A few detailed comments:

- 1st paragraph: previous evidence - please cite reviews on topic (e.g. Viner *et al. *2020). ^1^
- Reduction in rates of infection AND delay of epidemic/shift of course of epidemic.
- There is also a lot of literature on previous undesirable impacts of school closures, e.g. Cauchemez 2009 ^2^ and Aledort 2007 ^3^ which should be cited

**Methods:**

 This seems like a very reasonable and pragmatic approach to creating a logic model. Two specific comments:

- I am only wondering what types of studies you focused on (and whether that was an inclusion/exclusion criterion).
- I am interested in how you derived from a factor, which was integrated into a (quantitative) study as a variable, at a code that would then lead to a category represented in the logic model? Would it have to be a statistically significant association in order to be represented in the model?
- What qualified as a moderator? How did you determine whether a factor was considered a moderator rather than, e.g., a precondition or something else?
- There is some literature out there on how to derive at frameworks (e.g. determinant frameworks) which you could use to back up your approach

**Results:**

- Figure: Maybe consider creating a "smaller" graphical representation of the framework for easier access; I like the Miro Canvas, but the PPT figure is not readable; maybe reduce the level of granularity and only present the higher level domains. 
- The intervention: what about hybrid models as implemented in some countries in order to reduce the number of students in a class?
- Impacts on teachers and other school staff: there is some literature on this topic now, e.g. in the French and Israeli context; I would maybe consider thinking about additional need of staff (e.g. more staff is needed for facilitating hybrid forms of teaching as implemented in Germany; some teachers will not be able to teach in person in hybrid models).
- Age of students: it might be worthwhile to point out the differing presentations of disease in different age groups already in the result section (as you do in the discussion section).
- Social/political factors as moderators: maybe this should be referred to as context since it not only refers to social/political aspects but also to geographic, socio-economic and other factors.
- I am also wondering if the infrastructure in schools (e.g. the setup of classrooms, the possibility for outdoor teaching; cafeterias; rooms for teachers; etc.) plays a role when deciding whether or not to close/reopen schools.

**Future development:**

- It would be interesting to continue working on this model while evidence evolves  (e.g. by integrating causality as it arises) which the authors point out in the discussion.
- It would moreover be interesting to also discuss the question (and maybe pointing towards the literature on it) around re-opening; I am aware that the authors touch base on this, but I think more thought about the utility of the framework for thinking about under what circumstances schools can be reopened; what criteria have to be considered when making the decision; which stakeholders should be integrated in making decisions around school closure/opening; what plans have to be in place in order to facilitate reopening? I know that there is a lot we do not know yet, but maybe some food for thought?

Is the work clearly and accurately presented and does it cite the current literature?

Yes

If applicable, is the statistical analysis and its interpretation appropriate?

Not applicable

Are all the source data underlying the results available to ensure full reproducibility?

Yes

Is the study design appropriate and is the work technically sound?

Yes

Are the conclusions drawn adequately supported by the results?

Yes

Are sufficient details of methods and analysis provided to allow replication by others?

Yes

Reviewer Expertise:

Public Health; Framework synthesis; school closures during Corona; Qualitative research; logic model development

I confirm that I have read this submission and believe that I have an appropriate level of expertise to confirm that it is of an acceptable scientific standard.

## References

[1] : School closure and management practices during coronavirus outbreaks including COVID-19: a rapid systematic review. *The Lancet Child & Adolescent Health*.2020;4(5) : 10.1016/S2352-4642(20)30095-X 397-404 10.1016/S2352-4642(20)30095-X PMC727062932272089

[2] : Household transmission of 2009 pandemic influenza A (H1N1) virus in the United States. *N Engl J Med*.2009;361(27) : 10.1056/NEJMoa0905498 2619-27 10.1056/NEJMoa0905498 20042753PMC3840270

[3] : History of haemophilia. *Haemophilia*.2007;13 Suppl 5: 10.1111/j.1365-2516.2007.01576.x 1-2 10.1111/j.1365-2516.2007.01576.x 18240391
